# Supplementary material for: The genome sequence of the Flame Shoulder, Ochropleura plecta (Linnaeus, 1761)
Source: Wellcome Open Res. 2023 Nov 7;8:506. [Version 1] doi: 10.12688/wellcomeopenres.20187.1 (PMC10884592; doi:10.12688/wellcomeopenres.20187.1)
Supplement: Peer review history

# Reviewer report: Reviewer response for version 1

**DOI:** 10.21956/wellcomeopenres.22350.r74459

**Published:** 2024-03-07

**Authors:** Camille Meslin

**Affiliations:** 1 Institute of Ecology and Environmental Sciences of Paris, Université Paris Diderot, Versailles, France

The authors are presenting the genome assembly and annotation of the moth species Ochropleura plecta. The gathering of the data and genome assembly is well described. **The assembly is of good quality, being assembled into 32 chromosomal pseudomolecules, including the sex chromosomes. Overall, this new genome will be a great resource for the community. **However, **minor changes could improve the paper. In particular, **the annotation part could be completed. What was used exactly to conduct the annotation with Braker2? The authors mentioned RNA extraction in the methods, was it used in the annotation process? Also some sort of metrics for the genome annotation quality would be interesting. The authors have really good BUSCO statistics for the genome, it would be nice to compare them with the BUSCO for the annotation.

Are sufficient details of methods and materials provided to allow replication by others?

Partly

Is the rationale for creating the dataset(s) clearly described?

Yes

Are the datasets clearly presented in a useable and accessible format?

Yes

Are the protocols appropriate and is the work technically sound?

Yes

Reviewer Expertise:

chemical ecology, evolution, transcriptomics, genomics

I confirm that I have read this submission and believe that I have an appropriate level of expertise to confirm that it is of an acceptable scientific standard, however I have significant reservations, as outlined above.

# Reviewer report: Reviewer response for version 1

**DOI:** 10.21956/wellcomeopenres.22350.r72354

**Published:** 2024-02-22

**Authors:** Yi-Ming Weng

**Affiliations:** 1 Department of Entomology, University of Wisconsin Madison, Madison, WI, USA

According to the description of nucleic acid extraction and sequencing procedures, the transcriptomic data should have been generated. But the RNA reads were not used in coding gene prediction in BRAKER2 pipeline, I wonder if there is the quality issue or other reasons that the gene prediction did not involve transcriptomic data. i think it would be better to briefly explain the reasons.

Are sufficient details of methods and materials provided to allow replication by others?

Yes

Is the rationale for creating the dataset(s) clearly described?

Yes

Are the datasets clearly presented in a useable and accessible format?

Yes

Are the protocols appropriate and is the work technically sound?

Yes

Reviewer Expertise:

Entomology, Evolutionary Genomics, Population Genetics

I confirm that I have read this submission and believe that I have an appropriate level of expertise to confirm that it is of an acceptable scientific standard.

# Reviewer report: Reviewer response for version 1

**DOI:** 10.21956/wellcomeopenres.22350.r72352

**Published:** 2024-02-13

**Authors:** Huai-Jun Xue

**Affiliations:** 1 Nankai University, Tianjin, China

The authors have assembled the genome of the Flame Shoulder, *Ochropleura plecta* (Linnaeus, 1761) with Pacific Biosciences HiFi, 10×Genomics and Hi-C data. The genome size is 643.9 Mb with a scaffold N50 of 21.9 Mb, and the BUSCO completeness is 98.9% using the lepidoptera_odb10 reference set. This paper provided the information necessary to repeat this work.

 Legend of figure 1: If “ilOchPlec1” is a specimen name, it should be put after “specimen” other than the scientific name “ *Ochropleura plecta*”.

Are sufficient details of methods and materials provided to allow replication by others?

Yes

Is the rationale for creating the dataset(s) clearly described?

Yes

Are the datasets clearly presented in a useable and accessible format?

Yes

Are the protocols appropriate and is the work technically sound?

Yes

Reviewer Expertise:

Entomology, adaptation and speciation

I confirm that I have read this submission and believe that I have an appropriate level of expertise to confirm that it is of an acceptable scientific standard.
